# Neurosurgical Consequences of e-Scooter Use: Strategies to Prevent Neurological Injury

**DOI:** 10.1089/neur.2022.0073

**Published:** 2023-04-10

**Authors:** William McKay, William Kerscher, Muhammad Riaz, Alexander Mason

**Affiliations:** ^1^ Department of Neurosurgery, University of Colorado Anschutz Medical Campus School of Medicine, Aurora, Colorado, USA.; ^2^ Emory University, Denver, Colorado, USA.

**Keywords:** e-scooter, neurosurgery, public transportation, traumatic brain injury

## Abstract

Rideshare electric scooter accidents have led to increasing emergency department (ED) visits and neurosurgical consultations. This study categorizes e-scooter-related injuries requiring neurosurgical consultation at a single level 1 trauma center. Patients who required neurosurgical consultation from June 2019 to June 2021 with a positive finding on computed tomography imaging were selected for review of patient and injury characteristics, resulting in a sample size of 50 cases. Average patient age was 36.9 (15–69) years, and 70% were male. Seventy-four percent of patients were under the influence of alcohol and 12% illicit drugs. None (0%) were helmeted. Seventy-eight percent of accidents occurred between 6:00 pm and 6:00 am. Twenty-two percent of patients required surgical intervention by craniotomy/craniectomy, and 4% required intracranial pressure monitor placement. Average intracranial hemorrhage volume was 17.8 cc (trace to 125). Volume of hemorrhage was associated with the need for an intensive care unit (ICU) stay (odds ratio [OR] = 1.01; *p* = 0.04), need for surgical intervention (OR = 1.007; *p* = 0.0001), and mortality (1.816; *p* < 0.001) and trended toward, but did not reach significance for, overall poor outcome (OR = 1.63; *p* = 0.06). Sixty-two percent of this patient pool required ICU admission. Average length of ICU stay was 3.5 days (0–35), and average length of hospital stay was 8.3 days (0–82). Mortality in this series was 8%. Lower admission Glasgow Coma Scale (OR = 0.974; *p* < 0.001) and increased volume of hemorrhage (OR = 1.816; *p* < 0.001) were associated with increased risk of mortality in the linear regression analysis. Electric scooters have become prevalent in most urban centers, and accidents are a potential source of severe intracranial injury requiring extended ICU and hospital stays, surgical intervention, and sometimes resulting in long-term morbidity and/or mortality. Injuries often occur in the evening hours and are often associated with alcohol/drug use and lack of helmet use. Policy changes to help mitigate the risk of these injuries are recommended.

## Introduction

Rideshare electric scooters (e-scooters) have become commonplace in many urban centers since they were first established in Santa Monica, California in 2017. e-scooters have a top speed of around 15 mph and are designed to be used as microtransportation for commuters or tourists, who can download a mobile app and unlock the scooter for use on a minute-by-minute payment agreement. Some see this micromobility movement as a game changer in terms of transportation equity, allowing persons a cheap, flexible, and accessible alternative to standard public transportation options. Although e-scooter rideshare companies do provide a desired and needed service for some, there have been concerns since their implementation about misuse and injury. It has recently been well documented that e-scooters have become a contributing source to increasing emergency department (ED) visits and hospital admissions. Between 4% and 40% of scooter-related ED patients have been reported to have intracranial hemorrhage (ICH).^1–16^ The overall admission rate for scooter-related injuries is reported to be between 6% and 28%^2,10,14,15,17–21^ and intensive care unit (ICU) admission between 0.8% and 12%.^2,3,6,14,16,19–21^ In addition, the most common reason for admission to the hospital, and for intensive care, is for intracranial injury (ICI).^2,6,8,14,16,19,20^

One case series of 13 patients has been published detailing various cases requiring neurosurgical consultation, including ICH, central cord syndrome, and vertebral body compression fractures.^22^ There have otherwise been sparse data describing the neurosurgical consequences of e-scooter-related injuries. Given that traumatic brain injury (TBI)/ICI is often the most severe consequence of e-scooter-related injury, we reviewed all cases at our level 1 trauma center requiring neurosurgical consultation with a goal of further characterizing these injuries. In what we believe to be the largest case series to date, we describe our findings and frame our recommendations in the context of public health concerns and strategies to balance the benefits of e-scooter use with safety.

## Methods

### Data acquisition

**FIG. 1.**
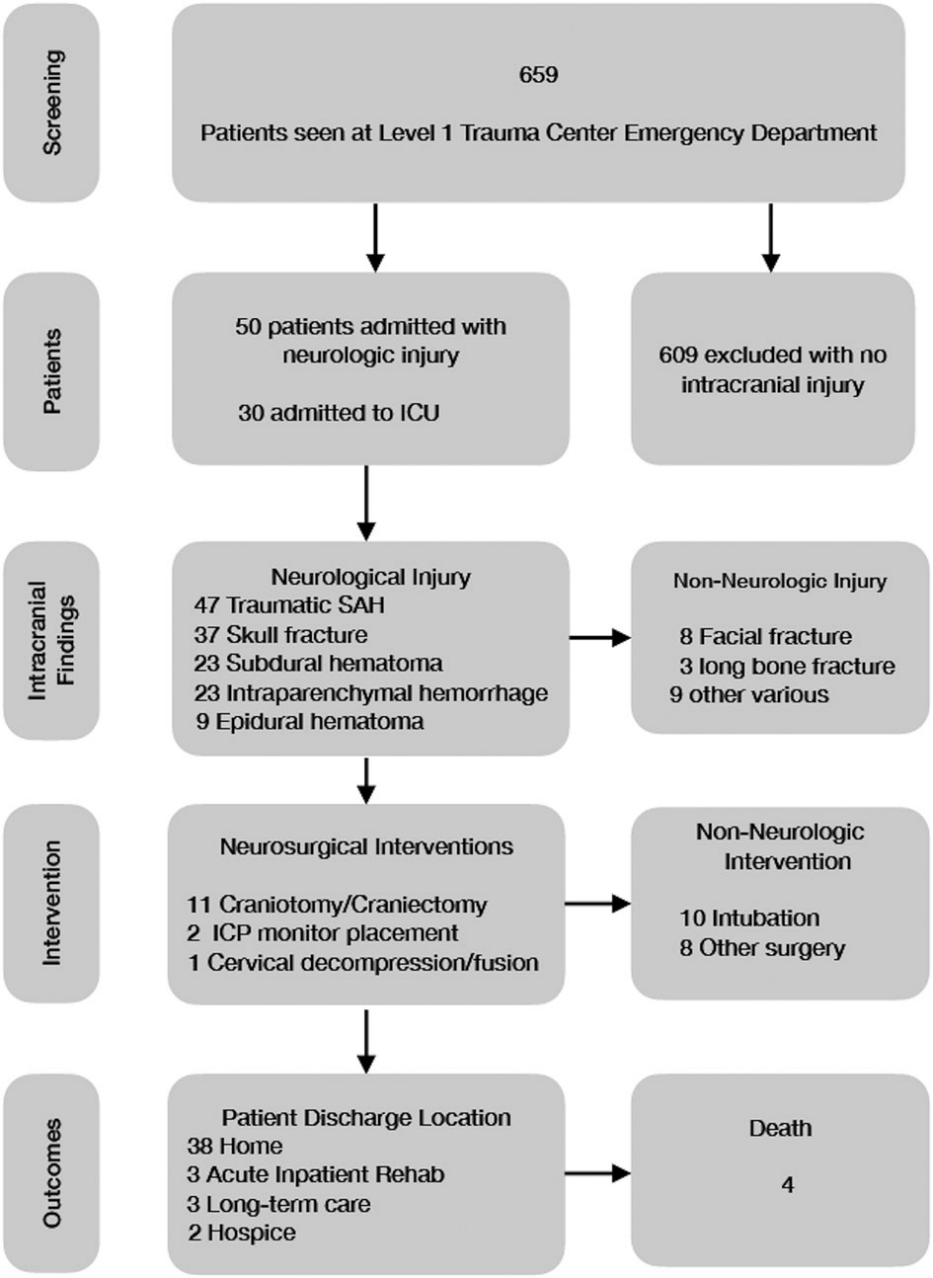
Patient selection and outcomes. ICU, intensive care unit; SAH, subarachnoid hemorrhage.

The institutional electronic medical record database was queried for all e-scooter-related encounters from June 2019 through June 2021 and reconciled with a prospective list maintained by the neurosurgery service. Those patients who required neurosurgical consultation with a positive finding reported by radiology on computed tomography imaging were selected for review (Fig. 1). The electronic medical record was then reviewed to determine demographic and clinical data as follows: patient age; sex; time of ED evaluation; helmet status; loss of consciousness (LOC); presence of anticoagulants or coagulation abnormalities; type of ICI; volume of ICH; admission Glasgow Coma Scale (GCS); whether ethanol or illicit drugs had been used; and blood alcohol level, if available.

Outcome data included whether surgery was required for intracranial pathology, other injuries sustained and whether surgery was required for those injuries, length of ICU stay, length of hospital stay, discharge location, discharge GCS, Glasgow Outcome Scale (GOS), discharge modified Rankin Scale (MRS), GOS/MRS at most recent follow-up, and mortality within 30 days. A good outcome was determined to be a GOS of 4 or 5 at discharge or the most recent follow-up, with poor outcome GOS 1–3. ICH volume was estimated using computer-assisted volumetry. The decision to pursue surgery was made by the attending neurosurgeon on call based on their clinical judgment.

### Statistical analysis

This is primarily a descriptive analysis. Means and medians with interquartile ranges were used to summarize continuous data. Percentages were used for the remaining frequency data. Linear regression was performed for continuous variables, and logistic regression for outcome variables, including mortality and good (GOS of 4–5) versus poor (GOS 1–3) functional outcome. Results are reported in odds ratios (ORs) and 95% confidence intervals. Statistical analysis performed with SPSS data analysis software (SPSS, Inc., Chicago, IL).

## Results

### Patient demographics

**FIG. 2.**
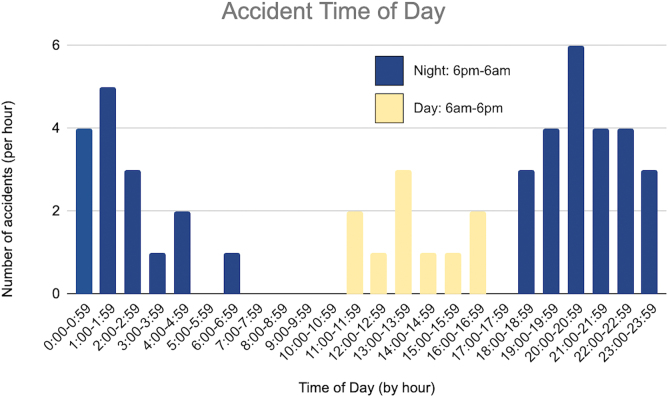
Accident time of day.

Over the study period from June 1, 2019 to June 30, 2021, there were 659 patients seen at our ED for e-scooter-related injuries (Table 1). Of those, 50 (7.6%) required neurosurgical consultation for ICI. Average age was 36.9 (15–69) years at the time of injury. Male patients made up 70% of the study population. The majority of patients were under the influence of alcohol (74%) and/or illicit drugs (12%), with an average blood alcohol level of 155 mg/dL. None were helmeted. Thirty-nine (78%) accidents occurred in the twilight hours between 6:00 pm and 6:00 am (Fig. 2). Forty-six (92%) were isolated scooter accidents, whereas the remaining four (8%) were automobile versus scooter accidents. None of the patients were taking anticoagulants; however, 1 patient with large-volume ICH requiring multiple repeat operations presented with anticoagulation abnormalities and was found to have autoimmune dysfunction of factor VIII.

**Table 1.** Patient Demographics

Demographic		
Sex	Total	Percent of total
Female	15	30
Male	35	70
Age (years)		
<18	1	2.0
18–25	8	16
26–40	27	55
41–65	12	24
>66	1	2.0
Time of accident		
12:00 am to 6:00 am	15	30
6:01 am to 12:00 pm	3	6
12:01 pm to 6:00 pm	9	18
6:01 pm to 11:59 pm	23	46
Intoxication		
Ethanol	37	74
Cocaine/amphetamines	4	8
Other illicit drugs	6	12

### Characterization of injuries

Forty-four (88%) patients experienced LOC at the time of injury, though all but 7 (14%) had regained consciousness by arrival to the ED (Table 2). Average GCS was 13 on arrival to the ED. Ten (20%) patients required intubation for low or declining GCS and inability to protect the airway. Eleven (22%) patients required surgical intervention by craniotomy/craniectomy for ICI, 2 (4%) required intracranial pressure (ICP) monitor placement, and 1 (2%) required a cervical spine decompression and fusion for spinal cord injury.

**Table 2.** Accident Characteristics

Characteristic		
Helmet use	*n* = 50	%
Yes	0	0
No	50	100
Loss of consciousness		
Yes	44	88
No	6	12
Admission Glasgow Coma Score		
3–6	3	6
7–10	7	15
11–15	38	79
Type of intracranial injury		
Traumatic subarachnoid hemorrhage	47	94
Skull fracture	37	74
Intraparenchymal contusions	23	46
Subdural hematoma	23	46
Epidural hematoma	9	18

Volume of intracranial hemorrhage	Average (mL)	Median (mL)
Volume (cc)	17.796	4.45

Patients presented with a variety of intracranial findings, often with multi-focal injury. The most common finding was traumatic subarachnoid hemorrhage (94%), followed by skull fracture (74%), subdural hematoma (46%), intraparenchymal contusions (46%), and epidural hematoma (18%). Average volume of ICH was 17.8 cc (trace to 125). Volume of hemorrhage was associated with the need for an ICU stay (OR = 1.01; *p* = 0.04), need for surgical intervention (OR = 1.007; *p* = 0.0001), and mortality (1.816; *p* < 0.001) and trended toward, but did not reach significance for, overall poor outcome (OR = 1.63; *p* = 0.06).

Twenty-five (62.5%) patients in our study group sustained concurrent non-neurological injuries, most commonly facial (40%) and long-bone (15%) fractures. Other injuries included vertebral compression fracture, unstable cervical spine ligamentous injury, blunt cerebrovascular injury, pulmonary contusion, rib fracture, and laceration to the liver/spleen/pancreas. Eight (20%) patients required non-neurosurgical operative intervention.

### Admission/length of stay

Of the 31 (62%) patients requiring ICU admission, average length of stay was 3.5 days (0–35), with a median of 2. Average length of hospital stay was 8.3 days (0–82), with a median of 4. There were 2 patients with a long clinical course and difficult social situations who were still boarding as inpatients at the time of this review.

Of the 45 surviving patients at the time of our review, 38 (76%) patients were able to discharge home at the end of observation/hospitalization. Three (6%) patients were transferred to an acute inpatient rehabilitation facility, 3 (6%) to a long-term acute care facility, and 1 (2%) to hospice.

### Morbidity/mortality

Four (8%) patients died of their e-scooter-related injuries (Table 3). Average discharge GCS was 14 (3–15), with a mode of 15. Average GOS on discharge was 4.5 (mode 5), and whereas most did not have a documented follow-up, those who did had an improvement by the most recent follow-up. Similarly, average MRS at discharge was 2 (median and mode of 1) and had improved by most recent follow-up, if performed. Lower admission GCS (OR = 0.974; *p* < 0.001) and increased volume of hemorrhage (OR = 1.816; *p* < 0.001) were associated with increased risk of mortality in the linear regression for GCS and volume of hemorrhage, but there were too few instances of mortality and poor outcome to find any statistically significant predictor of mortality or poor outcome among the factors studied in our logistic regression.

**Table 3.** Outcome Measures: GOS

Outcome measure		
Intensive care unit length of stay		Range
Average	3.54	0–35
Hospital length of stay		
Average	8.26	1–82

Poor outcome (GOS 1–3)	*n* = 50	%
GOS 1	5	10
GOS 2	0	0
GOS 3	4	8

Good outcome (GOS 4–5)		%
GOS 4	8	16
GOS 5	33	66
Mortality		
Mortality within 30 days	4	8

GOS, Glasgow Outcome Score.

## Discussion

The visibility, popularity, and use of e-scooters has continued to rise since their introduction, with average daily rides within the urban area served by our trauma center increasing from 4800 at the beginning of our study period to 6700 by the end.^26^ Given this increase, it is important to acknowledge the consequences of injuries associated with scooter use. To the best of our knowledge, the current study represents the largest characterization of neurosurgical injuries associated with e-scooter trauma to date. In support of previous literature on the subject, we found that intracranial neurological injury was the most common reason for admission or observation after e-scooter injury at our institution. The need for ICU care was 62%, much higher than the rate for the overall e-scooter injury population, which highlights the severity of neurological injuries in this patient population. Most injuries were non-operative and would be considered mild TBI. The long-term consequences of mild TBI, however, are often more significant than expected. Though we had little long-term follow-up in our series, we know from the existing literature that 33% of patients with mild TBI are functionally impaired and 82% have at least one symptom of post-concussion syndrome at 1 year after injury.^23^

Several patients in our series sustained severe TBI and required neurosurgical intervention (craniotomy and/or ICP monitor placement). These patients often had severe functional impairments, long hospital stays, and need for placement in long-term care facilities. The cost to the patient—emotional, financial, and physical—is significant. In addition, the costs to the healthcare system are substantial, with lifetime direct and indirect costs for moderate-to-severe TBI reported to be approximately $76.5 billion.^24^ The vast majority (90%) of total medical TBI costs are attributed to TBIs that require hospitalization or are fatal. To that note, there were several mortalities from scooter injuries in our series, which highlights the potentially serious consequences of this mode of transportation, which is generally thought of as benign.

In regard to the risk factors of injury, none in our study wore helmets, which is consistent with literature on the topic. A large majority (76%) in our series were under the influence of alcohol or drugs. This is higher than the reported rate in the overall e-scooter injury population, which has been reported as between 4.8% and 46%.^3,6,7,8,10–12,14,17,19,21,25^ The rate is consistent, however, with series that focus only on craniofacial trauma, which may indicate that intoxication may predispose one to these more severe cranial injuries. Nearly all (80%) injuries occurred in the evening or at night, which is also consistent with the overall e-scooter literature. Given those common risk factors and the fact that neurological injuries are the most severe and potentially fatal result of e-scooter use, we support policy changes that would mitigate these high-risk contributing factors. Suggested changes include: 1) requiring a breathalyzer be attached to the scooter to allow it to be unlocked; 2) attaching a helmet with a sensor that prevents the scooter from starting if the helmet is not worn, or requiring users to take a picture with the helmet in place and upload to the app before use; and/or 3) limiting use during night-time hours. We did not have available data on how fast the scooters were moving at the time of injury, but limiting speed may also prevent more severe injuries.

Of those suggestions, limiting use during the night-time hours and further limiting speed seem the most easy to implement, and there are existing technology and app-based software to accomplish those goals. There is evidence to suggest that most persons underestimate their level of intoxication and fitness to drive or, in this case, ride an e-scooter, making built-in breathalyzers a helpful preventative measure.^27^ Likewise, helmets have been shown to decrease TBI rates by 48% and serious TBI rates by 60% in cyclists.^28^ Though there are general differences between e-scooters and bicycles, the potential mechanisms of injury are similar and the protective benefit of helmets cannot be denied. Breathalyzers and mandatory helmet use would be much more difficult measures to implement, given that to our knowledge the technology we have described does not yet exist for the e-scooters currently being used. Updating the scooter technology to include those elements would likely have cost- and time-related as well as compliance effects that would make implementation difficult for rideshare companies. Despite those difficulties, we urge policy makers and e-scooter rideshare companies to consider the serious consequences we have described and work toward a solution that can prevent such injuries in the future.

### Limitations

This study was performed at a single level 1 trauma center, which could limit the results based on geographical and population constraints. It is limited by its retrospective nature and relatively small sample size. The study focused on relatively significant injuries requiring neurosurgical consultation, which may exclude the less severe injuries that might be seen at other level 1, 2, or 3 trauma centers. It is also limited by the lack of long-term follow-up for many patients, which hinders our ability to evaluate the eventual outcome for these patients. Given the low number of patients with poor outcome or mortality, the statistical analysis for these outcomes is not conclusive.

## Conclusion

Electric scooters have become a commonplace mode of transportation in most urban centers. They are a potential source of severe ICI requiring extended ICU and hospital stays, surgical intervention, and sometimes long-term morbidity and/or mortality. These injuries are often associated with modifiable risk factors, including alcohol and/or drug use, lack of helmet use, and use during the later hours of the night. We support policy changes to mitigate these factors to reduce the neurological injuries associated with e-scooter use.

## Abbreviations Used

ED: emergency department
GCS: Glasgow Coma Scale
GOS: Glasgow Outcome Scale
ICH: intracranial hemorrhage
ICI: intracranial injury
ICP: intracranial pressure
ICU: intensive care unit
LOC: loss of consciousness
MRS: modified Rankin Scale
OR: odds ratio
TBI: traumatic brain injury
